# Selective cytoprotective effect of histamine on doxorubicin-induced hepatic and cardiac toxicity in animal models

**DOI:** 10.1038/cddiscovery.2015.59

**Published:** 2015-12-21

**Authors:** DJMartinel Lamas, MB Nicoud, HA Sterle, E Carabajal, F Tesan, JC Perazzo, GA Cremaschi, ES Rivera, VA Medina

**Affiliations:** 1 Laboratory of Radioisotopes, School of Pharmacy and Biochemistry, University of Buenos Aires, Junín 956 PB, Buenos Aires, Argentina; 2 Laboratory of Cellular and Molecular Biology, Institute for Biomedical Research (BIOMED), School of Medical Sciences, Pontifical Catholic University of Argentina (UCA), and the National Scientific and Technical Research Council (CONICET), Buenos Aires, Argentina; 3 Neuroimmunomodulation and Molecular Oncology Division, Institute for Biomedical Research (BIOMED), School of Medical Sciences, Pontifical Catholic University of Argentina (UCA), and the National Scientific and Technical Research Council (CONICET), Buenos Aires, Argentina; 4 Department of Pathophysiology, School of Pharmacy and Biochemistry, University of Buenos Aires, Buenos Aires, Argentina

## Abstract

The aim of the present work was to evaluate the potential protective effect of histamine on Doxorubicin (Dox)-induced hepatic and cardiac toxicity in different rodent species and in a triple-negative breast tumor-bearing mice model. Male Sprague Dawley rats and Balb/c mice were divided into four groups: control (received saline), histamine (5 mg/kg for rats and 1 mg/kg for mice, daily subcutaneous injection starting 24 h before treatment with Dox), Dox (2 mg/kg, intraperitoneally injected three times a week for 2 weeks) and Dox+histamine (received both treatments). Tissue toxicity was evaluated by histopathological studies and oxidative stress and biochemical parameters. The combined effect of histamine and Dox was also investigated *in vitro* and *in vivo* in human MDA-MB-231 triple-negative breast cancer model. Heart and liver of Dox-treated animals displayed severe histological damage, loss of tissue weight, increased TBARS levels and DNA damage along with an augment in serum creatine kinase-myocardial band. Pretreatment with histamine prevented Dox-induced tissue events producing a significant preservation of the integrity of both rat and mouse myocardium and liver, through the reduction of Dox-induced oxidative stress and apoptosis. Histamine treatment preserved anti-tumor activity of Dox, exhibiting differential cytotoxicity and increasing the Dox-induced inhibition of breast tumor growth. Findings provide preclinical evidence indicating that histamine could be a promising candidate as a selective cytoprotective agent for the treatment of Dox-induced cardiac and hepatic toxicity, and encourage the translation to clinical practice.

## Introduction

Cancer is a major public health concern worldwide. Overall, there were 14.1 million new cases and 8.2 million deaths in 2012.^1^

Radiation and chemotherapy are widely used treatments for cancer. Despite their antitumoral effects controlling the primary tumor and metastasis, both therapeutic modalities can produce toxicity to normal tissues and frequently, their related adverse effects outweigh clinical benefits and worsen patient's quality of life.^2,3^

The anthracycline doxorubicin (Dox) is a highly effective anti-neoplastic agent, which intercalates in DNA and inhibits topoisomerase II. Dox is one of the most commonly systemic treatments to improve several adult and also pediatric cancers, including both hematological and solid tumors.^4–6^ Unfortunately, its clinical efficacy of Dox is hampered by dose-related toxicities, such as hematopoietic suppression and hepatotoxicity; although the most serious side effect is the life-threatening cardiomyopathy. The onset of cardiotoxicity may be delayed and become evident years after finalizing chemotherapy.^3,4,6^ Multiple cytotoxic mechanisms are involved in the pathogenesis of Dox-induced cardiotoxicity. However, a large body of evidence indicates that Dox-induced oxidative stress remains the cornerstone, as evidence by reactive oxygen species (ROS) induced oxidative damage such as lipid peroxidation.^3,4,6^ In addition, hepatotoxicity represents a common and severe side effect, in which oxidative stress also has a pivotal role.^7,8^

At present, there are no specific and effective therapeutic agents for Dox-associated cardio- or hepatotoxicity. Thus, the study of compounds that could improve the therapeutic index of chemo- and radiotherapy, reducing their side effects on healthy tissues without affecting their anti-neoplasic effects, is urgently needed.^3,9–11^ In this regard, previous data demonstrate that histamine administration was safely used in different experimental models as a radioprotective agent of normal radiosensitive tissues, including small intestine, salivary glands and bone marrow.^12–15^

Furthermore, histamine acts as an anti-proliferative agent in different cancer types. It was reported that histamine and histamine H4 receptor (H4R) agonists inhibit proliferation of two human breast cancer cell lines *in vitro* and *in vivo*, augmenting tumoral apoptosis.^16–21^ Similar anti-tumor effects of H4R agonists were observed in three different human melanoma cell lines^22^ and in cholangiocarcinoma experimental models.^23^

It is important to highlight that histamine not only exhibits anti-tumor effects but also potentiates the ionizing radiation action in breast cancer cells. Histamine produces a radiosensitizing action involving enhanced radiation-induced oxidative DNA damage and apoptosis and increasing DNA double-strands breaks. Furthermore, histamine was able to enhance *in vivo* the effect of gamma radiation, augmenting the exponential tumor doubling time of triple-negative breast cancer (TNBC) developed in nude mice.^18,24^

Based on these evidences, the aim of the present work was to evaluate the potential protective effect of histamine on Dox-induced hepatic and cardiac toxicity in different rodent species and in a triple-negative breast tumor-bearing mice model.

## Results

### Histamine reduces Dox-induced cardiotoxicity in rats

It is well known that a major adverse side effect associated with Dox clinical usage is the onset of cardiomyopathy.^3,4,6^ Cardiotoxicity was evaluated by both histopathological studies and oxidative stress and biochemical parameters. Serum creatine kinase-myocardial band (CK-MB) and aspartate aminotransferase (AST) are considered excellent markers for cardiac injury and are used to evaluate Dox-cardiotoxicity in different experimental models.^6,25^

**Figure 1:**
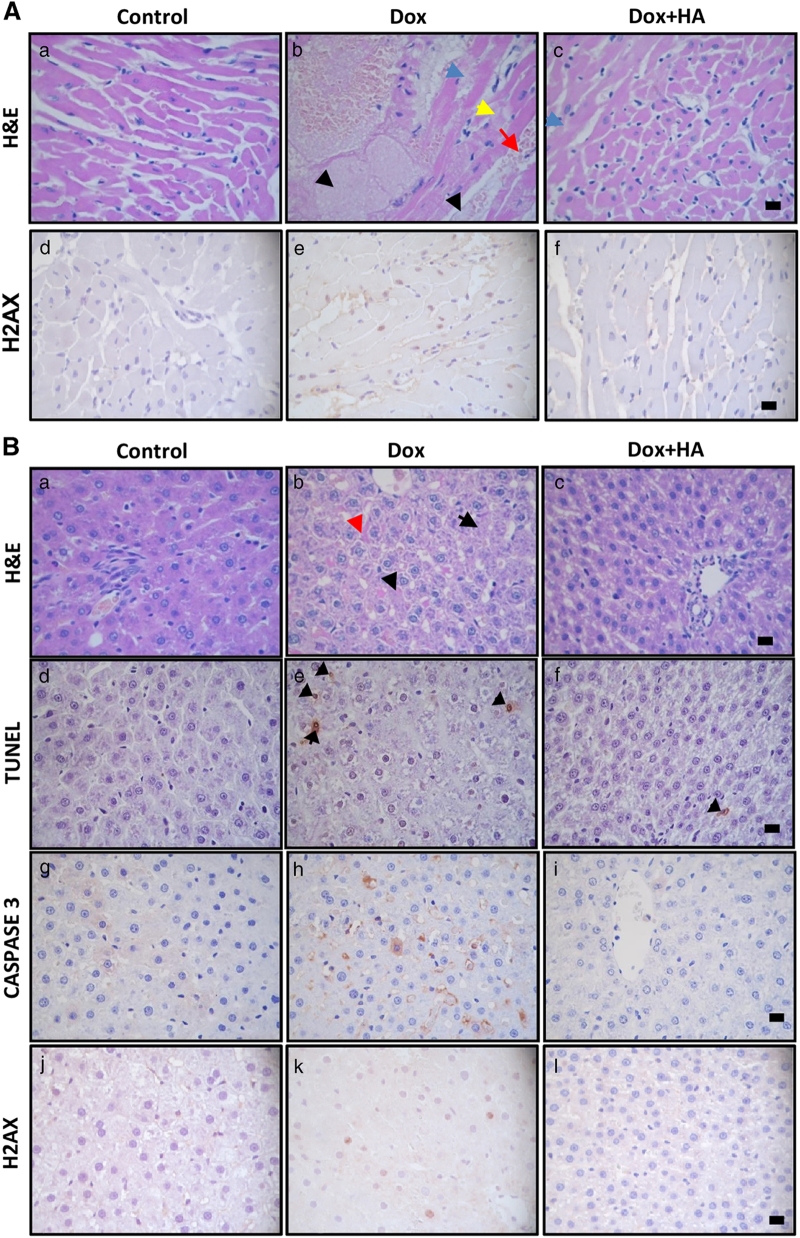

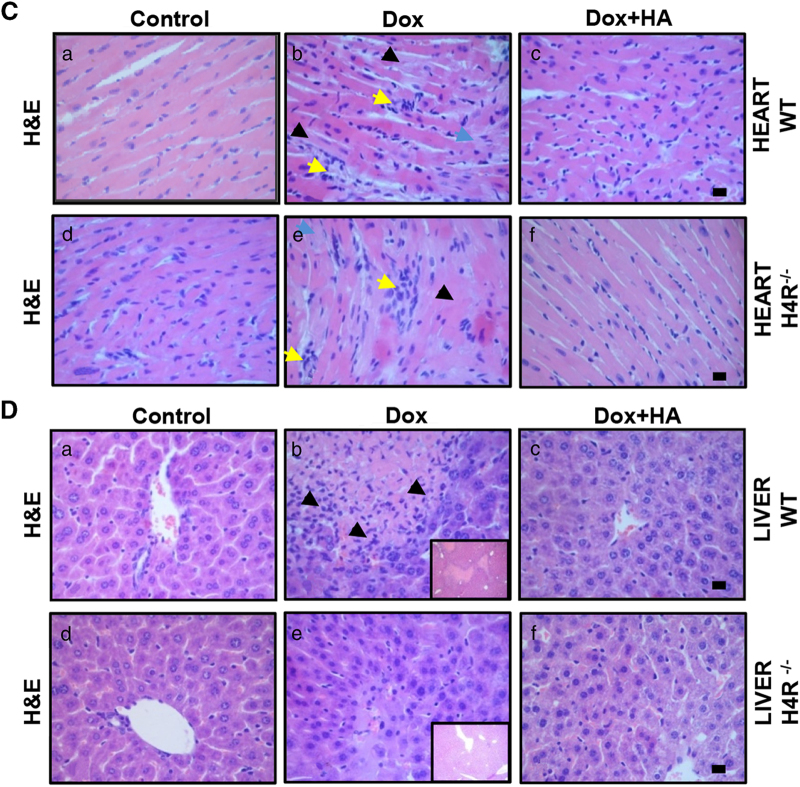
Histamine decreases doxorubicin-induced cardiotoxicity and hepatotoxicity in rats and mice. (**A**) (a, d) Normal histological appearance of untreated heart. (b, e) Heart of Dox-treated animals displaying focal necrotic cell death (black arrow), congestion-hemorrhage (red arrow), myocytolysis (yellow arrow) and myofribrillolysis (blue arrow) with fibrils de-arrangement. (c, f) Heart of Dox+HA-treated animals showing evident preservation of heart structure with reduced myofibrillolysis (blue arrow) and muscle bands with normal appearance. (a–c) Representative H&E stained specimens and (d–f) immunohistochemical images of *γ*H2AX sections are shown. (**B**) (a, d, g, j) Normal histological appearance of liver from untreated animals. (b, e, h, k) Liver of Dox-treated rats displaying de-arrangement of hepatic trabecula (red arrow), cellular edema and focal necrosis (black arrow). (c, f, i, l) Liver of Dox+HA-treated animals showing preservation of tissue structure with mild cellular edema. (a–c) Representative H&E-stained sections are shown. (d–f) Representative immunohistochemical images of TUNEL, (g–i) caspase 3 and (j–l) *γ*H2AX in paraffin-embedded liver specimens. Arrows indicate TUNEL-positive cells. (Six to eight rats per group). (**C**) (a) Normal histological appearance of WT mice untreated heart. (b) Heart of Dox-treated animals displaying vascular damage with perivascular edema, myofribrillolysis (blue arrow), cell recruitment (yellow arrows) and focal necrosis (black arrows). (c) Heart of Dox+HA-treated animals showing minimal focal damage with muscle bands with normal appearance. (d) Normal histological appearance of untreated heart of H4R^−/−^ mice. (e) Heart of Dox-treated H4R^−/−^ mice displaying myofibrillolysis (blue arrow), cell mobilization (yellow arrows) and focal necrosis (black arrow). (f) Heart of Dox+HA-treated H4R^−/−^ mice showing minimal focal damage (non-diffused), including rippled and non-extended myofibrillolysis, with vasculature with normal appearance. (**D**) (a) Normal histological appearance of untreated liver of WT mice. (b) Liver of Dox-treated animals displaying two different areas, an extended necrotic region and a normal area, between them cellular mobilization as a band (arrow) (c) Liver of Dox+HA-treated WT mice showing almost normal characteristics. (d) Normal histological appearance of liver of untreated H4R^−/−^ mice. (e) Liver of Dox-treated H4R^−/−^ displaying reduced histological damage with focal and non-diffuse necrotic areas without cell mobilization. (f) Liver of Dox+HA-treated H4R^−/−^ showing preservation of hepatic structure. Representative H&E stained sections are shown. x630 Original magnification. Scale bar, 20 *μ*m. Inset: image at x100-fold magnification. (8–12 Mice per group).

The heart of Dox-treated rats showed a severe histological damage with congestion, rippled myocytes, reduction of striated muscle bands, hemorrhagic areas, myocytolysis and focal necrosis, along with enhanced expression of DNA damage marker *γ*H2AX, effects that were markedly reduced by histamine administration (Figure 1A).

**Figure 2:**
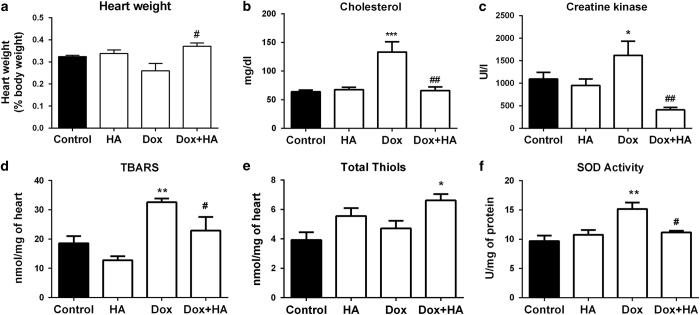
Histamine blocks doxorubicin-induced cytotoxic and oxidative damage in rat’s heart. (**a**) Heart weight determined as percentage of body weight. (**b**) Serum cholesterol levels. (**c**) Serum CK-MB levels. (**d**) TBARS levels expressed as nmol/mg of cardiac tissue. (**e**) Thiols content expressed as nmol/mg of tissue. (**f**) SOD activity expressed as U/mg of heart proteins. (Six to eight rats per group, *P<0.05, **P<0.01, ***P<0.001 *versus* control; ^#^ *P*<0.05, ^##^ *P*<0.01 *versus* Dox).

Dox-induced cardiotoxicity was also manifested by an increase in CK-MB and cholesterol levels and a decrease in the ratio of heart weight to body weight (Figures 2a–c). In addition, myocardial tissue from Dox-treated rats showed significant increases in TBARS production and SOD levels (Figures 2d and f).

On the other hand, histamine significantly alleviated the increase in lipid peroxidation and SOD activity, as well as serum CK-MB activity and blocked the decrease in heart weight while enhanced thiol levels in Dox-treated rats (Figures 2a–f). Non-significant changes were observed in catalase activity (Supplementary Table S1) and unexpectedly a significant decrease in AST levels were observed in both Dox and Dox+HA groups in rats and mice (Supplementary Table S2) as it was previously observed.^25^

### Histamine ameliorates Dox-induced hepatotoxicity in rats

**Figure 3:**
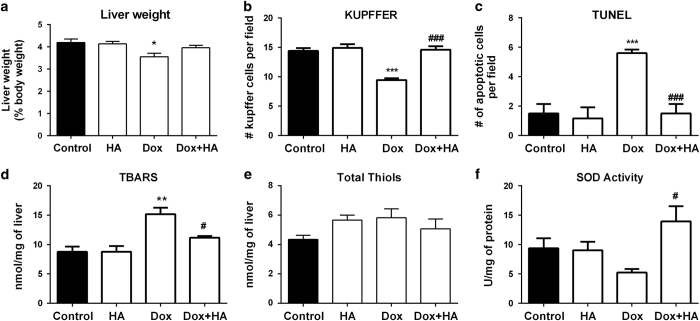
Histamine alleviates doxorubicin-induced cytotoxic and oxidative damage in a rat's liver. (**a**) Liver weight determined as percentage of body weight. (**b**) The number of Kupffer cells and (**c**) the number of TUNEL-positive cells were determined by counting 10 random fields. (**d**) TBARS levels expressed as nmol/mg of cardiac tissue. (**e**) Thiols content expressed as nmol/mg of tissue. (**f**) SOD activity expressed as U/mg of liver proteins. (Six to eight rats per group, *P<0.05, **P<0.01, ***P<0.001 *versus* control; ^#^ *P*<0.05, ^###^ *P*<0.001 *versus* Dox).

Dox produced drastic cellular abnormalities in the liver, including focal necrosis and fibrosis, sinusoidal atrophy and edema and reduced liver weight (Figures 1B and 3a). Dox also significantly increased apoptosis, which was evaluated by the TUNEL assay and activated caspase 3, and also phosphorylation of H2AX in the liver of treated rats (Figures 1B and 3c). The combined treatment with histamine markedly preserved hepatic weight and tissue structure, which only showed mild vacuolization (Figures 1B and 3a), reduced DNA damage and the number of apoptotic cells, exhibiting similar characteristics to the untreated liver (Figures 1B and 3c).

Kupffer cells are long-lived tissue macrophages located in sinusoids with a pronounced endocytic and phagocytic capacity and important host-defense functions.^26^ Dox significantly diminished the number of Kupffer cells in hepatic lobules, effect that was blocked with histamine treatment (Figure 3b). In addition, a significant increase in lipid peroxidation was observed in the liver of Dox-treated rats, while histamine treatment prevented this effect (Figure 3d). Non-significant changes were observed in hepatic thiol content, while the combination of histamine and Dox increased SOD activity (Figures 3e and f). Both histamine and Dox administration increased hepatic catalase activity (Supplementary Table S1). No evident changes were observed upon histamine administration (Figures 3a–f).

### Histamine reduces cardiac and hepatic toxicity in wild-type and H4R^−/−^ mice

The former described experiments suggested that histamine produced cytoprotective effects on Dox-induced toxicity in rat heart and liver. To confirm its protective effects, another animal experimental model of Balb/c mice was further investigated.

**Figure 4:**
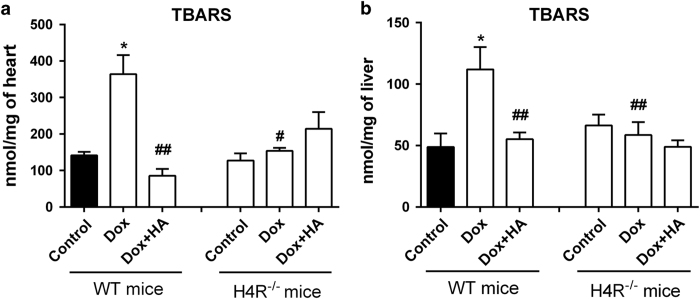
Doxorubicin and histamine effects on TBARS levels of H4R^−/−^ mice compared with WT Balb/c mice. TBARS levels were determined in mice (**a**) heart and (**b**) liver of WT and KO mice. Data are expressed as nmol/mg of tissue. (8–12 mice per group, *P<0.05 *versus* WT Control; ^#^ *P*<0.05, ^##^ *P*<0.01 *versus* WT Dox).

Hearts of Dox-treated WT mice exhibited the expected histopathological abnormalities, including focal necrosis and vascular damage that were accompanied by a significant increase in cardiac lipid peroxidation compared with saline-treated control mice (Figures 1C and 4a). Histamine markedly reduced histopathological changes, preserving muscle bands and vasculature and also blunted the Dox-induced rise in TBARS levels (Figures 1C and 4a).

In agreement with previous data, Dox exhibited a severe hepatotoxic effect in Balb/c mice, which was evidenced by many areas of necrosis surrounded by cellular mobilization, and enhanced TBARS levels (Figures 1D and 4b). As it was shown in rat model, histamine prevented both hepatic histological and oxidative damage in Dox-treated mice (Figures 1D and 4b).

Interestingly, H4R^−/−^ mice were less affected by Dox-induced histopathological alterations and lipid peroxidation in heart and liver compared with WT mice (Figures 1C and D and 4a and b). Nevertheless, histamine administration was able to prevent the manifested histological damage in heart and liver of Dox-treated H4R^−/−^ mice (Figures 1C and D).

### Histamine enhances Dox-induced anti-proliferative effects on MDA-MB-231 triple-negative breast cancer cells

**Figure 5:**
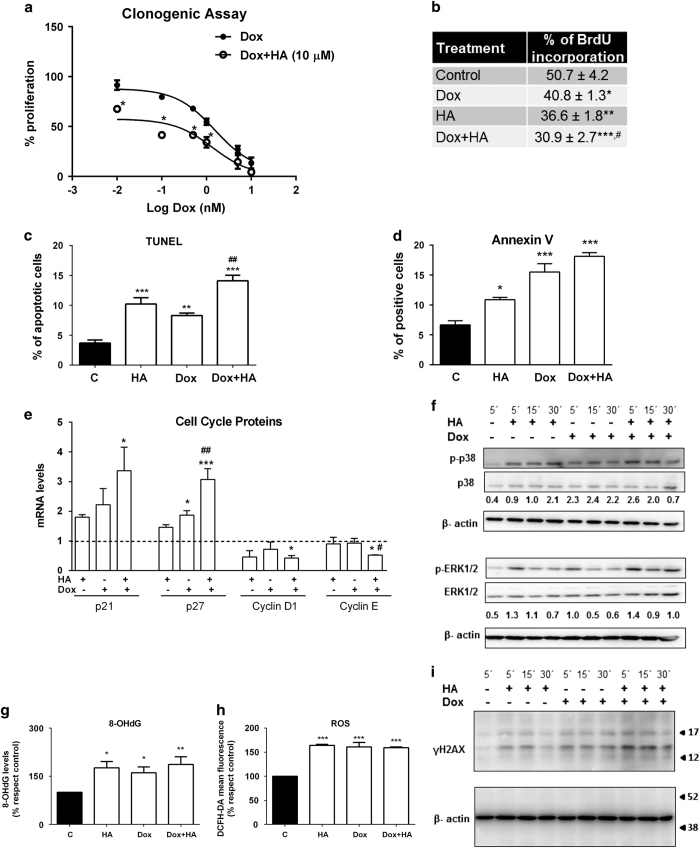
Histamine enhances anti-proliferative properties of doxorubicin *in vitro*. (**a**) Proliferation was evaluated by the clonogenic assay in human TNBC MDA-MB-231 cells treated with Dox (0.01–10 nM) in the absence (closed circles) or presence (open circles) of 10 *μ*M histamine. Proliferation was expressed as a percentage relative to untreated cells (*n*=3, *P<0.01 *versus* Dox; two-way ANOVA and Bonferroni post test). (**b**) Incorporation of BrdU, (**c**) TUNEL and (**d**) Annexin-V staining assays were evaluated in MDA-MB-231 cells that were left untreated (control; C) or were treated with histamine (HA, 10 *μ*M) and/or doxorubicin (Dox, 10 nM) for 48 h. (**e**) The mRNA expression levels of p21, p27, cyclin D1 and cyclin E2 were determined 24 h after treatments using qPCR and the expression levels were normalized to the expression of *β*-2-microglobulin. The ΔΔCt method was used to calculate the fold change. (**g**) Oxidative DNA damage was evaluated by measuring 8-OHdG formation and (**h**) intracellular ROS levels were determined 24 h after HA and/or Dox treatments using flow cytometry. (*n*=3-5, *P<0.05, **P<0.01, ***P<0.001 *versus* control; ^#^ *P*<0.05, ^##^ *P*<0.01 *versus* Dox). Time course effects of Dox and HA on (**i**) *γ*H2AX (15 kDa) and (**f**) phospho-MAPKs (p-ERK1/2, 42/44 kDa and p-p38) were assayed by western blot. Total ERK1/2, p38 and *β*-actin (42 kDa) were used as loading control. Semiquantitative analyses of band intensities are shown (*n*=2).

To determine whether histamine could affect the anti-tumoral effect of Dox and considering that this chemotherapeutic agent is one of the first-line treatments in TNBC,^27^ the combined effect of histamine and Dox on proliferation in MDA-MB-231 cells was first investigated. Clonogenic assay demonstrated that both single agents induced a dose dependent inhibition on the proliferative capacity of MDA-MB-231 TNBC cells^16^ and histamine (10 *μ*M) increased Dox inhibitory effect (Figure 5a). According to the calculated CI using the Chou-Talalay method,^28^ Dox and histamine combination showed synergistic anti-tumoral activity (CI<1) tested at a 50% effective dose, calculated at Dox (5 nM) and histamine (10 *μ*M) (CI=0.41) or Dox (10 nM) and histamine (10 *μ*M) combinations (CI=0.16).

The incorporation of BrdU was further evaluated as a measurement of active proliferation. The 48 h combined treatment of Dox and histamine resulted in a modest, but significant, additional reduction of the incorporation of BrdU compared with Dox used alone (Figure 5b).

Consistently, both TUNEL and Annexin V assays show a significant increase in the rate of apoptotic cell death in Dox-treated cells, effect that was enhanced by the combined treatment with histamine (Figure 5c and d).

Furthermore, the combined treatment of Dox and histamine downmodulated cyclin D1 and E2 mARN levels, whereas upregulated the expression of p27 (Kip1) and p21 (Figure 5e).

Dox may exert its anti-neoplastic effect, in part, by causing DNA damage^5^ and 8-OHdG is a major type of oxidative DNA damage marker.^29^ Results demonstrate that both single agents and the drug combination increased 8-OHdG formation (Figure 5g). In addition, single treatments alone induced phosphorylation of H2AX, a marker of DNA double-strand breaks,^24^ while the combination treatment enhanced DNA damage further (Figure 5i).

Dox was reported to induce ROS generation in several tumor cells.^4,6^ Dox, histamine and the combination treatment increased ROS production (Figure 5h).

Similar effects were observed in the hormone-dependent MCF-7 breast cancer cells, in which Dox reduced proliferation dose dependently and the combined treatment with histamine enhanced this inhibitory effect on cell growth, while also increased Dox-induced ROS levels (Supplementary Table S3).

The mitogen-activated protein kinase such as extracellular signal-regulated kinase (ERK) and p38 are involved in cell growth, death and breast cancer chemoresistance.^30^ Therefore, Dox and histamine effects on phospho-ERK1/2 (p-ERK1/2) and phospho-p38 (p-p38) were assayed by western blot. Both compounds induced the phosphorylation of ERK1/2 and p38 in MDA-MB-231 cells (Figure 5f).

### Histamine induces a selective effect on doxorubicin cardiac and hepatotoxicity in triple-negative breast cancer-bearing mice

**Figure 6:**
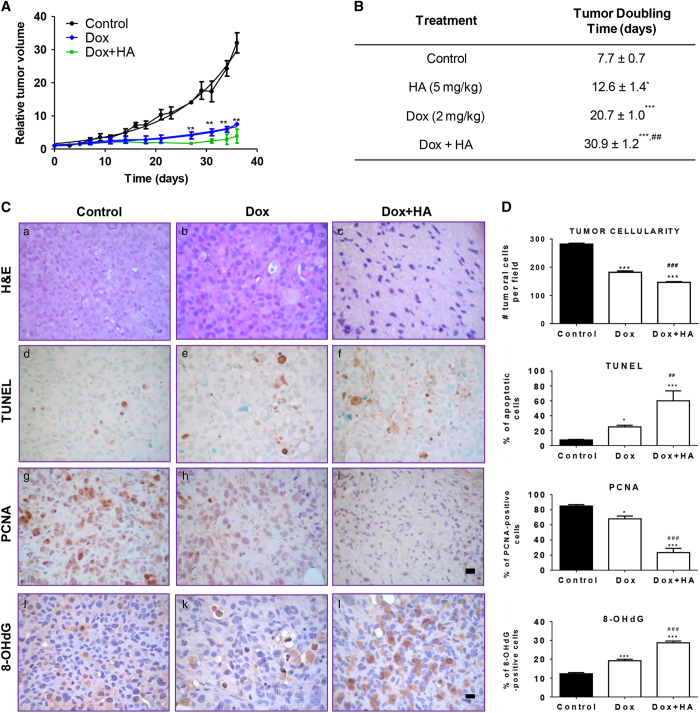

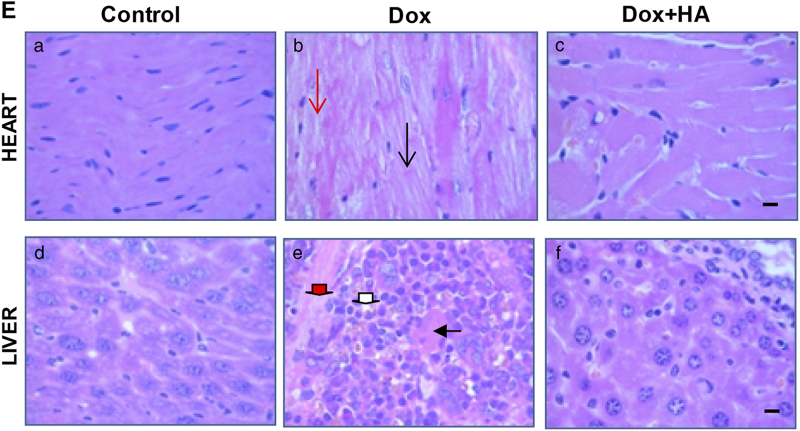
Combined effect of histamine and doxorubicin on triple-negative breast tumors induced in nude mice. (**A**) Relative tumor volume of the control group *versus* doxorubicin (Dox, 2 mg/kg) or the combination of Dox (2 mg/kg) and histamine (HA, 5 mg/kg). (6 mice per group, **P<0.01 *versus* Control; *T*-Test). (**B**) Median tumor doubling time of each group is depicted numerically (*P<0.05, ***P<0.001 *versus* Control; ^##^ *P*<0.01 *versus* Dox. *T*-Test). (**C**) Histopathological and immunohistochemical analyses of tumor tissues. (a–c) Representative H&E-stained sections are shown. (a) Untreated tumors presented undifferentiated adenocarcinoma cells with marked anisokaryosis and anisocytosis. (b) Dox increased necrosis and the nuclear optical density. (c) The combined treatment Dox+HA reduced tumor cellularity, with evident nuclear hyperchromasia, that was replaced by extracellular matrix. Representative immunohistochemical images of (d–f) TUNEL, (g–i) PCNA and (j–l) 8-dOHG in paraffin-embedded tumor tissues. x630 Original magnification. Scale bar, 20 *μ*m. (**D**) The number of tumor cells and the percentage of TUNEL, PCNA and 8-OHdG-positive stained cells were quantified by counting 10 random fields. (*P<0.05, ***P<0.001 *versus* Control; ^##^ *P*<0.01, ^###^ *P*<0.001 *versus* Dox). (**E**) Representative H&E stained sections of heart and liver are shown. (a) Normal histological appearance of untreated heart. (b) Heart of Dox-treated animals displaying severe myocytolysis (red arrow), areas of necrosis (black arrow) and reduced striated muscle bands. (c) Heart of Dox+HA-treated animals showing preservation of the structure with reduced myocytolysis, nuclei and muscle bands with normal appearance. (d) Normal histological appearance of untreated liver. (e) Liver of Dox-treated animals displaying focal necrosis (black arrow), sinusoidal atrophy (white arrow), inflammatory infiltrates, and fibrosis (red arrow). (f) Liver of Dox+HA-treated animals showing reduced sinusoidal disarrangement, displaying similar characteristics of the untreated liver. x1000 Original magnification. Scale bar, 20 *μ*m.

The combination therapy of Dox and histamine was further studied *in vivo* in a human TNBC model induced in nude mice by injection of MDA-MB-231 cells. Dox significantly reduced tumor size and increased doubling time, while the combined treatment of Dox and histamine enhanced significantly Dox-mediated decrease in the rate of proliferation (Figures 6A and B).

Histopathological analysis shows that Dox decreased the number of mitosis per field and cellularity. The combined treatment of Dox and histamine almost completely decreased mitosis per field and further diminished tumor cellularity that was replaced by extracellular matrix (Figures 6C and D).

Consistently, histamine potentiated Dox-induced increased tumoral apoptosis and reduced PCNA proliferation marker expression, while increasing DNA oxidative damage evaluated by 8-OHdG formation (Figures 6C and D).

As it was demonstrated in the other experimental models described, Dox produced severe cardiotoxicity in nude mice evidenced by numerous areas of myocytolysis and necrosis. Again, histamine administration counteracted the deleterious effect of Dox on heart, reducing histological damage (Figure 6E).

Dox also produced marked hepatic histological alterations, including sinusoidal atrophy and fibrosis, effects that were alleviated by the combination with histamine (Figure 6E).

## Discussion

Dox is one of the most effective anticancer drugs, but its clinical use is limited by life-threatening cardiotoxicity. Apart from its therapeutic cytotoxic effect on cancer cells through interacting DNA, Dox-induced ROS formation and oxidative damage. Both effects are particularly important in the pathogenesis of cardiac and hepatic injury.^3–5,10^ At present, no clinically proven treatment is established for Dox-induced cardiomyopathy. Therefore, novel approaches and the development of safe chemoprotective drugs against detrimental effects of Dox on normal tissues without hindering its anti-tumor activity are of upmost importance together with their translatability to clinical practice.^3,4,10^

In the present study we show that Dox produces severe histopathological changes in heart of Sprague Dawley rats and also Balb/c mice, effects that are accompanied by a marked increase in cardiac lipid peroxidation, SOD activity and DNA damage and in serum CK-MB and cholesterol levels and also by a decrease in heart's weight, findings similar to those in other studies.^3,31–33^ Although lipid peroxidation and CK-MB level are undoubtedly increased, several studies have reported no significant change, increase or decrease activities of SOD and catalase, the major enzymes participating in free radical metabolism, depending on Dox concentration, intervals of administration, time of evaluation or experimental model.^4,31^

Remarkably, pretreatment with histamine prevented all Dox-induced tissue events producing a significant preservation of both rat and mouse myocardium integrity, likely through a reduction of Dox-induced oxidative stress. This hypothesis is reinforced by our previous works, which demonstrated the protective effect of histamine on ionizing radiation-induced injury of different sensitive tissues through a modulation of antioxidant enzymes and reduction of genotoxic damage.^12–15^ Present data show, for the first time, the potential cytoprotective effect of histamine against Dox-induced cardiotoxicity by reducing oxidative stress and also DNA damage (by *γ*H2AX marker). In this regard, recent studies demonstrated that histamine through the activation of H3R produces significant protective effects, alleviating norepinephrine-induced arrhythmias that characterize myocardial ischemia/reperfusion (I/R).^34^ In addition, the H3R agonist imetit produces a cardioprotective action, improving isoproterenol-induced hemodynamic, plasma cardiac biomarkers, tissue antioxidant status and histopathology.^35^ On the other hand, activation of mast cell H4R, possibly by mast cell-derived histamine during I/R produces cardioprotective anti-renin angiotensin system effects with reduction of norepinephrine release, alleviating reperfusion arrhythmias.^36^

Hepatotoxicity is another frequent side effect of Dox chemotherapy with a significant impact on patients' outcomes.^7,8^ In agreement with previous data,^7,37^ Dox also increases hepatic lipid peroxidation and apoptosis, while reduces liver´s weight and SOD activity. These effects paralleled with serious histopathological alterations. Pretreatment with histamine significantly prevents all the evaluated Dox-induced toxic manifestations, preserving liver structure. Therefore, histamine represents an effective approach to reduce Dox-induced hepatotoxicity. In addition, histamine blocks the Dox-induced reduction of Kupffer cells, liver macrophages involved in the control of tumor growth and infection.^26,38^ Ongoing studies of dynamic hepatobiliary scintigraphy showed a reduced ^99m^Tc-disida extraction with Dox administration, effect that was blocked by histamine (FT, unpublished data), suggesting that histamine could prevent Dox-induced hepatic dysfunction.

In support to this hypothesis, other authors reported that histamine effectively protects liver against I/R-induced histological, functional and oxidative damage. Histamine effect was not blocked by pretreatment with mepyramine (H1R antagonist) or ranitidine (H2R antagonist) but was reversed by pretreatment with thioperamide (H3R and H4R antagonist).^39^ Furthermore, histamine effect was mimicked by treatment with clozapine,^39^ an anti-psychotic drug that is also considered not only H4R but also H3R agonist.^40^ Therefore, further studies using specific pharmacological blockade and/or genetically H4R knockdown system are needed to confirm the hepatoprotective role of H4R. In line with this data, treatment with clozapine (1 mg/kg, s.c.) reduced histological and oxidative stress injury in Dox-treated rats, although to a lesser extend compared to histamine treatment (DJML, unpublished data).

Importantly, we found that in heart and liver of H4R^−/−^ mice, non-significant increase in TBARS levels was observed after Dox administration and a reduced histological damage was shown especially in liver of Dox-treated animals, which exhibited diminished areas of necrosis. Nevertheless, histamine improved histological features in Dox-treated H4R^−/−^ mice, suggesting in the one hand that another histamine receptor might be involved in histamine cytoprotective effect and on the other hand, that H4R contributed to Dox-induced damage. Our next experiments will focus on the identification of the receptor subtype/s involved in histamine cytoprotection. In addition, considering the role of inflammation in chemotherapy-induced cytotoxicity^31^ and the anti-inflammatory properties of pharmacological H4R blockade in preclinical and clinical studies,^40,41^ we will investigate the effect of H4R antagonists to evaluate whether they could be beneficial to treat Dox side effects. In this regard, we previously demonstrated that pretreatment with the selective H4R antagonist JNJ7777120 reduced radiation-induced genotoxic, oxidative stress and histological damage on small intestine, salivary glands and hematopoietic tissues.^42^

Several protective agents have been investigated to prevent Dox-induced damage in preclinical models. However, none of the strategies has been translated into clinical practice.^4,10,25,31^ In addition, only some of the published approaches verified whether the treatments compromised Dox therapeutic efficacy. This anthracycline is one of the standards of care in TNBC, which accounts for 15–20% of all breast cancers and is characterized by poor prognosis.^27^ Thus, we investigated the combined effect of histamine and Dox in MDA-MB-231 TNBC cells. Our study demonstrated that histamine increased Dox-induced anti-tumoral activities not only *in vitro* but also *in vivo.* Histamine enhances Dox-induced apoptosis and DNA damage, while modulates p-ERK1/2 and p-p38 expression in MDA-MB-231 cells. Importantly, in the tumor-bearing mouse model we were able to reconfirm the selective cardio and hepatoprotective action of histamine.

It is important to highlight that histamine has been reported to be relatively a low-toxic compound, well tolerable by both animals of different species at similar or higher concentrations employed at the present study^39,43,44^ as well as cancer patients. Histamine is being used in clinical trials as an adjuvant to immunotherapy.^44–46^

Collectively, present findings indicate that histamine exhibits chemoprotective effects against Dox-induced cytotoxic and oxidative damage in heart and liver. Thus, the combined use of histamine with Dox could be an attractive strategy to improve the therapeutic ratio of Dox. Especially taking into account that histamine treatment also produces the inhibition of tumor growth and the induction of apoptosis without compromising the anti-tumor activity of Dox and exhibiting differential cytotoxicity. In conclusion, histamine could be a promising candidate as a selective cytoprotective agent for the treatment of cardiac and hepatic toxicity caused by Dox chemotherapy.

## Materials and methods

### Animals and treatments

Male Sprague Dawley rats (200–250 g) and Female athymic nude (NIH nu/nu) mice (20–25 g) were purchased from the Division of Laboratory Animal Production, School of Veterinary Sciences, University of La Plata, Buenos Aires, Argentina. Balb/c H4R knockout (H4R^−/−^, 6.129S5 tm1 [Histamine 4 Receptor] Lex) mice were gifted by Janssen Research & Development, LLC (NJ, USA) and Balb/c wild-type mice both were obtain from The Jackson Laboratory (Sacramento, CA, USA).

Animals (aged 8–10 weeks) were kept 4–6 per cage and maintained in our animal health care facility at 22 to 24 °C and 50–60% humidity on a 12 h light/dark cycle with food and water available ad libitum. Animal procedures were in accordance with recommendations from the Guide for the Care and Use of Laboratory Animals of the National Research Council, USA, and protocols were approved by the Ethical Committee for the Use and Care of Laboratory Animals of BIOMED (UCA-CONICET).

Doxorubicin (Dox) and histamine (HA) were freshly dissolved in saline solution. Animals were separated into four groups (*n*=6–12 each): control group, HA, Dox and Dox+HA. HA and Dox+HA groups received a daily subcutaneous (s.c.) HA injection for 2 weeks (1 mg/kg for BALB/c WT and KO mice and 5 mg/kg for rats) starting 1 day before the first dose of Dox (2 mg/kg) in Dox+HA group. Dox was administered every other day intraperitoneally (i.p.) in six injections for 2 weeks in Dox and Dox+HA groups. Control group received saline. Animals were sacrificed 1 day after the last dose of Dox. Tumor-bearing nude mice received both treatment (daily s.c. 5 mg/kg histamine injection and three i.p. injection per week of 2 mg/kg of Dox) until the end of the experimental period (5 weeks). Serum levels of CK-MB, aminotransferases and cholesterol were assessed from an intracardiac blood sample taken at the time of sacrificing the anesthetized animal ('Hospital de Clínicas', University of Buenos Aires, Buenos Aires, Argentina).

### Tumor development and growth evaluation

Tumors of MDA-MB-231 cells were developed as previously described.^16^ When the graft volumes reached 100–150 mm^3^, xenografted mice were separated in four groups and were treated until killing as described. To evaluate tumor growth the length and width of the subcutaneous tumors were measured using a caliper three times a week.^16,24^ The tumor size was calculated as sphere volume. Tumor growth data were expressed as relative tumor volume (tumor volume measured with respect to initial tumor volume at the beginning of treatment) and analysis was carried out using GraphPad Prism version 5.00. The equation for exponential growth was *Y_t_* =*Y*_0_xe^(*k*x*t*)^, where *Y*_0_ was the initial relative tumor volume that increased exponentially with a rate constant, *k*. The tumor doubling time was calculated as 0.69/k.

### Histopathological and immunohistochemical studies

Tissues and tumors were removed and were fixed with 10% neutral buffered formalin and after embedding in paraffin, specimens were cut into serial sections of 4 *μ*m thick. Histopathological characteristics were examined after hematoxylin-eosin staining (H&E).

Immunohistochemistry was performed as it was previously described.^17^ Briefly after blocking, tissues were incubated with primary goat anti-8-hidroxydeoxyguanosine (8-OHdG, 1:200, Millipore, Temecula, CA, USA), mouse anti-proliferating cell nuclear antigen (PCNA, 1:100, DAKOCytomation, Glostrup, Denmark), rabbit anti-phosphorilated histone H2AX antibody (*γ*H2AX, Cell Signaling Technology, Beverly, MA, USA), or rabbit anti-cleaved caspase 3 (Abcam, 1:100 Cambridge, MA, USA) antibodies overnight in a humidified chamber at 4 ºC. Immunoreactivity was detected by using Vectastain ABC Kit (Vector Laboratories INC., Burlingame, CA, USA) according to the manufactures’ instructions or with horseradish peroxidase-conjugated anti-goat antibody (1:250, Sigma Chemical Co., St Louis, MO, USA.) and visualized by diamino-benzidine staining (Sigma Chemical Co.).

Apoptotic cells were detected, as earlier reported,^12^ using ApoptagTM plus peroxidase *in situ* apoptosis Detection Kit (Millipore) according to the manufacturer's instructions.

Light microscopy was performed on an Axiolab Karl Zeiss microscope (Göttingen, Germany). All photographs were taken using a Canon PowerShot G5 camera (Tokyo, Japan). Specimens were assessed and scored to provide a quantitative measurement by using ImageJ, NIH software.

### Evaluation of total thiol content, TBARS levels and catalase and SOD activities

The thiobarbituric acid reactive species (TBARS) assay is a well-established method for screening and monitoring lipid peroxidation. The method used in the present study, was described by Yagi^47^ and adapted as previously reported.^42^ A molar extinction coefficient of *ε*=1.56×10^5^/M/cm was used for calculations.

Tissue total thiols concentration was estimated by the ability of the sulfhydryl group to reduce 5,5´-dithiobis(2-nitro-benzoic acid) (DTNB, Sigma-Aldrich) according to Tietze^48^ and was described previously by us.^42^ A molar extinction coefficient of *ε*=13.6/mM/cm was used for calculations.

Catalase activity was measured spectrophotometrically by monitoring the disappearance of H_2_O_2_ at 240 nm, as it was previously described.^42,49^ A unit of catalase was defined as the disappearance of 1*μ*mol of H_2_O_2_/min (*ε*=43.6/mM/cm).

Superoxide dismutase (SOD) activity was assayed by inhibition of adrenochrome formation rate at 480 nm. One unit of SOD is determined as the amount of enzymatic protein required to inhibit 50% epinephrine auto-oxidation.^42,49^

### Cell culture and proliferation assays

MDA-MB-231 and MCF-7 cells (American Type Tissue Culture Collection, Manassas, VA, USA) were were maintained and clonogenic assay was performed as previously reported.^16,24^ Cells were seeded in 6-well plates (1,200 cells/well) and were treated with Dox (0.01–1000 nmol/l) alone or with HA (0.01–10 *μ*mol/l) or remained untreated. Cells were incubated for 7 days and were then fixed and stained with 1% w/v toluidine in 70% v/v in ethanol. Cutoff were colonies containing 50 cells or more and data was expressed as a percentage of the untreated wells.

To examine the interaction between histamine and Dox, the isobolanalysis was employed and the combination index (CI) was determined according to the Chou-Talalay method using CompuSyn software (ComboSyn Inc, NJ, USA). The resulting CI theorem offers quantitative definition for additive effect (CI=1), synergism (CI<1), and antagonism (CI>1) in drug combinations.^28^

Quantification of cellular DNA synthesis was performed by 5-bromo-2′-deoxyuridine (BrdU, Sigma Chemical Co.) incorporation assay as previously described.^19^ Briefly, cells were treated with Dox (10 nM) and/ or HA (10 *μ*M) and were maintained up to 48 h after. BrdU (30 *μ*mol/l) was added to cultures the last 2 h. Cells were fixed and after denaturing the DNA, cells were incubated with anti-BrdU mouse monoclonal antibody (1:100, Sigma Chemical Co.) and then with horseradish peroxidase-conjugated anti-mouse IgG (1:100, Sigma Chemical Co.). Finally cells were visualized by diamino-benzidine staining (Sigma Chemical Co.) and light microscopy (Axiolab Karl Zeiss).

### Western blot analysis

Western blot analysis was performed as previously described.^24^ The primary antibodies were diluted as follows: mouse anti-phospho-ERK (p-ERK1/2, 1:500, Santa Cruz Biotechnology, Santa Cruz, CA, USA), mouse anti-phospho-p38 (p-p38, 1:500, 1:500, Santa Cruz Biotechnology), rabbit anti-ERK (ERK1/2, 1:1000, Santa Cruz Biotechnology), mouse anti-p38 (p38, 1:500, 1:500, Santa Cruz Biotechnology), rabbit anti-γH2AX (1:500, γH2AX, Cell Signaling Technology) and mouse anti-*β*-actin (1:1500, Aviva System Biology, San Diego, CA, USA). Immunoreactivity was detected by using horseradish peroxidase-conjugated anti-mouse or anti-rabbit as appropriate (Sigma Chemical Co.), and ECL system (Amersham ECL Prime western blotting detection reagent; GE Healthcare, Buckinghamshire, UK). Densitometric analyses were performed using the software ImageJ 1.32 J (NIH, Bethesda, MD, USA).

### Determination of apoptosis

Apoptotic cells after a 48 h treatment were determined by TdT-mediated UTP-biotin Nick End labeling (TUNEL) assay according to the manufacturer's instructions (CHEMICON International, CA, USA). Cells were visualized using Axiolab Karl Zeiss microscope (Göttingen, Germany).

Phosphatidylserine exposure on the surface of apoptotic cells was detected by flow cytometry after staining with Annexin V-FITC (BD biosciences, USA), and PI (50 *μ*g/ml). Data were analyzed using BD AccuriCSampler software (Becton Dickinson Co., Franklin Lakes, NJ, USA).

### Measurement of intracellular ROS production

After a 24 h treatment, cells were incubated with 5 *μ*mol/l dichlorodihydrofluoresceindiacetate (DCFH2-DA) (Sigma Chemical Co.) and ROS levels were measured by flow cytometry and data analysis was performed using BD AccuriCSampler software (Becton Dickinson Co.).

### Determination of 8-OHdG by flow cytometry

Cells were treated for 24 h and then washed, detached by trypsinazation, and were then fixed with methanol at −20 °C for 10 min. Fixed cells were treated with RNase (100 *μ*g/ml) for 1 h at 37 °C and proteinase K (10 *μ*g/ml) (Sigma Chemical Co.) for 10 min at room temperature. After rinsing with PBS, DNA was denatured by treatment with 4 nmol/l HCl for 10 min followed by pH adjustment with 50 mmol/l Tris (pH 10) for 5 min at room temperature. After blocking in 5% (w/v) equine serum in PBS, cells were incubated 30 min at RT with goat 8-OHdG (1:100, Millipore). Cells were washed with PBS and incubated for 30 min with 1:300 fluorescein isothiocyanate (FITC)-conjugated anti-goat Immunoglobuline G (IgG) and mean fluorescence was determined by flow cytometry and data analysis was performed using BD AccuriCSampler software (Becton Dickinson Co.).

### RT and real-time quantitative PCR (qPCR)

After a 24 h treatment, cells were removed and immediately homogenized in Trizol Reagent (Life Technologies Co., Carlsbad, CA, USA) to isolate the RNA, according to the manufacturer’s instructions. The RNA pellets were dissolved in RNase-free water and the RNA concentration was quantified by measuring the absorbance at 260 nm (NanodropND-1000, Thermo Fisher Scientific Inc., Wilmington, DE, USA). cDNA was synthesized by retrotranscription using the Omniscript kit (Qiagen, Valencia, CA, USA) following the manufacturer’ instructions using 2 *μ*g total RNA and 1 *μ*mol/l oligodeoxythymidine (Biodynamics SRL, Buenos Aires, Argentina). PCRs were performed using a commercial mastermix for real-time PCR containing SYBR Green fluorescent dye (Biodynamics SRL) in a total volume of 25 *μ*l, which contained 10 pmol of each primer and 1 *μ*l of cDNA and employing a RotorGene-6000 DNA thermal cycler (Corbett, Life Sciences, Sydney, NSW, Australia). The cycling conditions were 95 °C for 15 min, followed by 40 cycles of denaturation at 95 °C for 10 s, annealing at 60 °C for 15 s, and extension at 72 °C for 30 s. Primer sequences (Biodynamics SRL) were designed using the Primer Express Software version 3.0 (Applied Biosystems, Foster City, CA, USA) (Supplementary Table S4). Quantification of the target gene expression was performed using the comparative cycle threshold (Ct) method. An average Ct value was calculated from the duplicate reactions and normalized to the expression of *β*2-microglobulin. The ΔΔCt value was then calculated as previously reported.^50^

### Statistical analysis

Unless otherwise indicated, all data shown are mean±S.E.M. Statistical evaluations were made by analysis of variance that was followed by Newman–Keuls' Multiple Comparison Test unless otherwise indicated, using GraphPad Prism Version 5.00 software (San Diego, CA, USA). *P*-values <0.05 were considered statistically significant.

## Abbreviations

Dox: doxorubicin
ROS: reactive oxygen species
HA: histamine
H4R: histamine H4 receptor
TNBC: triple-negative breast cancer
CK-MB: creatine kinase myocardial band
AST: aspartate aminotransferase
SOD: superoxide dismutase
TBARS: thiobarbituric acid reactive species
WT: wild type
CI: combination index
BrdU: 5-bromo-2′-deoxyuridine
TUNEL: terminal deoxynucleotidyl transferase dUTP nick end labeling
8-OHdG: 8-hydroxy-2-deoxyguanosine
ERK: extracellular signal-regulated kinase
p-ERK1/2: phospho-ERK1/2
p-p38: phospho-p38
PCNA: proliferating cell nuclear antigen
H3R: histamine H3 receptor
I/R: ischemia/reperfusion
H1R: histamine H1 receptor
H2R: histamine H2 receptor
H4R−/−: H4R knockout
KO: knockout
i.p.: intraperitoneally
s.c.: subcutaneous
H&E: hematoxylin–eosin staining
DTNB: 5,5′-dithiobis(2-nitro-benzoic acid)
DCFH2-DA: dichlorodihydrofluoresceindiacetate
Ct: cycle threshold
PI: propidium iodide
RT: room temperature
IgG: immunoglobulin G
qPCR: quantitative PCR.
