# Supplementary material for: Selective cytoprotective effect of histamine on doxorubicin-induced hepatic and cardiac toxicity in animal models
Source: Cell Death Discov. 2015 Dec 21;1:15059–. doi: 10.1038/cddiscovery.2015.59 (PMC4979467; doi:10.1038/cddiscovery.2015.59)
Supplement: Supplementary Information [file cddiscovery201559-s1.doc]

Table 1 Supplementary Data

|  |  | **Control** | **HA** | | **Dox** | **Dox+HA** |
| --- | --- | --- | --- | --- | --- | --- |
| **Catalase Activity** | **Heart**  **Liver** | 32.6 ± 7.7  572.7 ± 22.5 | 26.6 ± 5.9  726.63 ± 15.9* | 28.7 ± 1.3  741.7 ± 13.9* | | 31.5 ± 6.1  681.5 ± 16.1* |

**Table S1: Effect of Dox and histamine on the levels of Catalase activity in rats´ heart and liver.** (*P<0.05 vs. Control).

Table 2 Supplementary data

|  |  | **Control** | **HA** | **Dox** | **Dox+HA** |
| --- | --- | --- | --- | --- | --- |
| **AST**  **level** | **Rat**  **Mice** | 186.7 ± 17.1  7.5 ± 2.5 | 143.0 ± 11.1  6.2 ± 1.7 | 91.3 ± 1.3**  3.5 ± 0.7** | 73.0 ± 9.8**  3.5 ± 0.7** |

**Table S2: Effect of Dox and histamine on serum AST levels.** (**P<0.01 vs. Control).

Table 3 Supplementary data

| **Treatment** | **% of BrdU incorporation** | **ROS production (% control)** |
| --- | --- | --- |
| Control | 41.3 ± 1.1 | 100 ± 3 |
| HA | 28.8 ± 1.8* | 144 ± 2*** |
| Dox | 30.3 ± 2.2* | 146 ± 2*** |
| Dox+HA | 17.2 ± 1.0***^,###^ | 212 ± 3***^,###^ |

**Table S3: Effect of histamine and Dox on cell proliferation and ROS production in MCF-7 cells.** MCF-7 cells were left untreated (Control) or were treated with histamine (10 μM) and/or Dox (10 nM) and proliferation was evaluated by BrdU incorporation while ROS formation was determined by DCFH-DA staining and flow cytometry 48 h after treatment. (n=3, *P<0.05,***P<0.001 vs. Control; ^###^P<0.001 vs. Dox).

Table 4 Supplementary data

| **Gene** | **Primer sequences** | **Tm (ºC)** |
| --- | --- | --- |
| *Cyclin D1*  *(Ccnd1)* | F- 5´-TCCTCTCCAAAATGCCAGAG-3´  R- 5´-TGAGGCGGTAGTAGGACAGG-3´ | 57.2  58.1 |
| *Cyclin E2*  *(Ccne2)* | F- 5´-TACTGACTGCTGCTGCCTTG-3´  R-5´-AAAAGTCTTCAGCTTCACTGGA-3´ | 58.8  58.2 |
| *p21/Cip1* | F-5´-CCATGTGGACCTGTCACTGT-3´  R-5´-GGATTAGGGCTTCCTCTTGG3´ | 59.2  58.8 |
| *p27/Kip1* | F-5´-TGCAGGTCGCTTCCTTATTC-3´  R-5´-CTAACTCTGAGGACACGCATT T-3´ | 59.9  58.2 |

**Table S4: Primer sequences**
